# Zika Virus Infection in Pregnant Women, Yucatan, Mexico

**DOI:** 10.3201/eid2508.180915

**Published:** 2019-08

**Authors:** Yamila Romer, Nina Valadez-Gonzalez, Silvina Contreras-Capetillo, Pablo Manrique-Saide, Gonzalo Vazquez-Prokopec, Norma Pavia-Ruz

**Affiliations:** Emory University, Atlanta, Georgia, USA (Y. Romer, G. Vazquez-Prokopec);; Universidad Autónoma de Yucatan, Yucatan, Mexico (N. Valadez-Gonzalez, S. Contreras-Capetillo, P. Manrique-Saide, N. Pavia-Ruz)

**Keywords:** Zika virus, viruses, infection, pregnancy, pregnant women, congenital abnormalities, vector-borne infections, Yucatan, Mexico

## Abstract

We report demographic, epidemiologic, and clinical findings for a prospective cohort of pregnant women during the initial phase of Zika virus introduction into Yucatan, Mexico. We monitored 115 pregnant women for signs of active or recent Zika virus infection. The estimated cumulative incidence of Zika virus infection was 0.31 and the ratio of symptomatic to asymptomatic cases was 1.7 (range 1.3–4.0 depending on age group). Exanthema was the most sensitive clinical sign but also the least specific. Conjunctival hyperemia, joint edema, and exanthema were the combination of signs that had the highest specificity but low sensitivity. We did not find evidence of vertical transmission or fetal anomalies, likely because of the low number of pregnant women tested. We also did not find evidence of congenital disease. Our findings emphasize the limited predictive value of clinical features in areas where Zika virus cocirculates with other flaviviruses.

## Abstract 2

Infection was associated with a high incidence of symptomatic disease but no congenital syndrome.

Zika virus, a mosquitoborne flavivirus, emerged abruptly in the Americas. It was first recognized in Brazil during 2015 in association with an outbreak of exanthematic disease, which was quickly linked to neurologic and immunological complications and congenital malformations (*1**–**6*). The first epidemic wave was centered in northeastern Brazil and associated with a high incidence of vertical transmission and cases of congenital disease that reached peaks of 49.9 cases/10,000 live births (*7*). The virus quickly spread to other countries and affected large sectors of Central America, South America, southern regions of North America, and the Caribbean (*8*). However, similarly high rates of congenital disease were not observed in other regions or in subsequent transmission waves in northeastern Brazil (*9**,**10*). The magnitude of the risk for vertical transmission and congenital syndromes, as well as possible associations that might increase or decrease these risks, remain unknown.

Multiple factors have been suggested to explain regional differences in disease incidence, including ethnic, environmental, nutritional, and virologic factors, as well as herd immunity (*11**–**14*). In addition, the possibility of overreporting of cases because of high public and epidemiologic awareness has been considered (*11**,**15*). The objectives of this study were to characterize the incidence, epidemiologic characteristics, clinical manifestations, and birth outcomes after Zika virus infection in pregnant women during the early phase of virus introduction in the state of Yucatan, Mexico.

## Methods

### Population

We have been evaluating integrated strategies to prevent *Aedes* mosquitoborne diseases in Yucatan State, Mexico. After health authorities confirmed the presence of Zika virus in Mexico, we designed a prospective study to quantify the incidence of disease and infection in pregnant women. The catchment area included a longitudinal cohort of 884 families (3,993 persons) residing in the cities of Merida, Ticul, and Progreso de Castro in Yucatan State (*16*). Merida and its metropolitan area, which have ≈1 million inhabitants, contain ≈50% of the Yucatan population. Progreso de Castro (population ≈37,400) and Ticul (population 32,000) are smaller urban areas. We enrolled consenting pregnant women from these areas during July 1, 2016–August 31, 2017. In addition, we independently enrolled pregnant women referred by physicians in primary care facilities or hospital facilities involved in our cohort study during the same period.

### Clinical Follow-up of Pregnant Women and Newborns

Patient monitoring included a monthly visit for clinical assessment and sample collection (blood and urine), weekly doctor follow-up by text messages, and complete access to a telephone to report any clinical signs in pregnant women, their newborns, or any family contact. Fetal ultrasonography was performed at enrollment and every 3 months. At the first visit, a questionnaire was given to establish the clinical–epidemiologic profile. The pregnancy follow-up ended when the pregnancy was completed by delivery or fetal loss or the participant withdrew from the study. After initial clinical evaluation (anthropometric measurements, APGAR score [*17*], and clinical complications) and sample collection from the newborn, the postnatal follow-up included an evaluation during the first 18 months of life to detect development of any anomalies. These evaluations included cognitive and psychomotor status, neurology, ophthalmology, and genetic and audiology >1 time during this period. We used a definition of microcephaly based on the recommendation of the World Health Organization (*18*); cranial circumference >2 SDs below the mean for the age and sex of the baby.

### Laboratory Testing

We detected Zika virus RNA by using real-time reverse transcription PCR (RT-PCR) for blood and urine samples as described (*19**–**22*). We also performed RT-PCR for Zika virus for blood of newborns and cerebrospinal fluid, as well as products of conception, including amniotic fluid, placenta, and fetal tissues, according to clinical needs (*23*). The RT-PCR studies were conducted in the Laboratory of Clinical Hematology of the Centro de Investigaciones Regionales (Merida, Mexico).

### Statistical Analysis

We compared clinical and epidemiologic variables between pregnant women who were infected with Zika virus during pregnancy and women who remained Zika virus–negative by RT-PCR by calculating odds ratios and testing for their significance by using the Fisher 2-sided exact test. We evaluated differences in head circumference between groups of babies born to Zika virus–positive and –negative mothers by using the Wilcoxon signed rank test. Differences in p-values <0.05 were considered statistically significant. All analyses were performed by using SPSS version 24 software (IBM, https://www.ibm.com).

## Results

A total of 115 pregnant women were included in the study: 66 from Merida, 45 from Ticul, and 4 from Progreso de Castro. One third were positive for Zika virus by RT-PCR of blood, urine, or both, at the initial evaluation (26 women) or during follow up (10 women). The cumulative incidence of Zika virus infection in the cohort was 0.31. The symptomatic to asymptomatic ratio among PCR-positive patients infected with Zika virus was 1.7 (range 1.3–4.0, depending on age group), and the highest proportion was in women 20–29 years of age (Table 1). Of the 26 positive patients at baseline, 22 had blood and urine samples, 3 had only blood samples, and 1 had only a urine sample. Of 22 paired blood and urine samples, 5 were Zika virus positive for both samples, 16 were Zika virus positive only for blood samples, and 1 was Zika virus positive only for a urine sample. Three unpaired blood samples and 1 unpaired urine sample were positive for Zika virus (Table 2).

**Table 1:** Symptomatic and asymptomatic Zika virus–positive pregnant women, by age group, Yucatan, Mexico*

Age group, y	Symptomatic	Asymptomatic	p value†	S:A Ratio
15–19	2	5	0.073	0.4
20–29	16	4	0.038	4
30–49	5	4	0.693	1.25
Total	23	13	<0.0001	1.7

*Patients were positive by PCR. A, asymptomatic; S, symptomatic. †By Fisher exact test.

**Table 2:** PCR results for pregnant women at time of first positive sample for Zika virus infection, Yucatan, Mexico

Result	No. (%)
Zika virus positive at time of enrollment, n = 26	
Blood and urine positive	5 (19)
Blood positive, urine negative	16 (61)
Blood positive without urine tested	3 (11)
Urine positive without blood tested	1 (4)
Blood negative, urine positive	1 (4)
Zika virus positive during follow-up, n = 10	
Blood and urine positive	3 (30)
Blood positive, urine negative	3 (30)
Blood positive without urine tested	1 (10)
Urine positive without blood tested	2 (20)
Blood negative, urine positive	1 (10
Total, n = 36	36 (100)
Blood and urine positive	8 (22)
Blood positive, urine negative	19 (53)
Blood positive without urine tested	4 (11)
Urine positive without blood tested	3 (8)
Blood negative, urine positive	2 (6)

In subsequent monthly testing, 11 (42%) blood samples remained positive and 2 urine samples that were negative in the first test became positive. In the third interval, 4 (15%) blood samples remained positive, and only 1 (3%) remained positive during the fourth interval. No urine sample was positive in 2 consecutive monthly controls. No intermittent urine virus shedding was detected. Seven (50%) of 14 women with virus shedding in urine had clinical symptoms at the time of virus detection. For 5 patients, the positive urine sample occurred at the same time as the positive blood sample, and for 2 patients, urine was positive after the blood sample showed a negative result. Of the 10 patients in whom infection developed during follow-up, 7 had paired blood–urine samples; 3 of those had positive blood and urine samples, 3 had only positive blood samples, and 1 had only a positive urine sample. Two patients were positive only for the urine sample, and 1 was positive only for the blood sample. For 1 patient, a urine sample negative at the time of detecting the infection became positive in the subsequent monthly control while the blood sample became negative. One patient had a blood sample that remained positive for >1 time interval.

We obtained the distribution of the cases per epidemiologic week for Yucatan State and the national epidemiologic curve (Figure, panel A). We detected cases of Zika virus infection 3 weeks before the passive surveillance system detected any cases, and time series of case counts in the cohort matched the epidemiologic curve for the passive surveillance system in shape and temporality (Figure, panel B). Of the Zika virus–positive mothers 8 were enrolled during the first trimester, 23 during the second trimester, and 5 during the third trimester. Of the Zika virus–negative mothers, 22 were enrolled during the first trimester, 39 during the second trimester, and 18 during the third trimester. Two weeks after the date of last menstruation was considered representative of the moment of conception and was established for 100 case-patients (33 positive for Zika virus and 67 negative for Zika virus).

**Figure:**
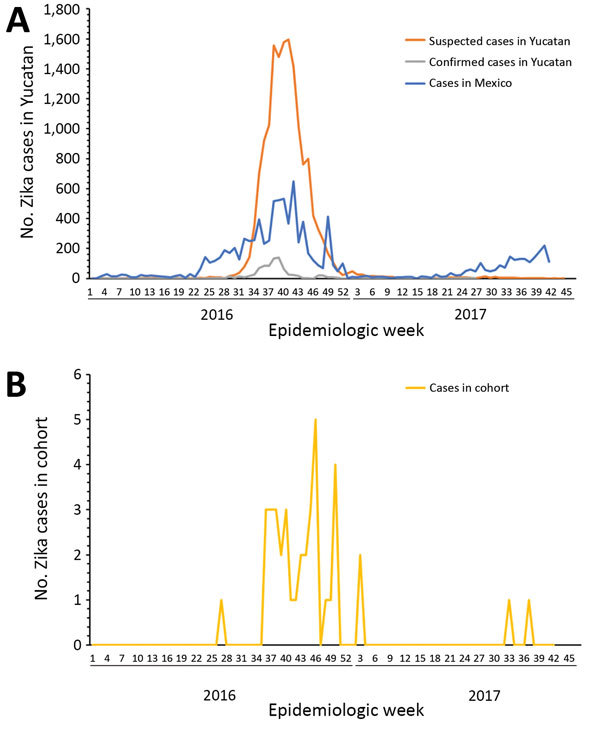
Distribution of cases of Zika virus infection, by epidemiologic week, Mexico, 2016–2017. A) Suspected and confirmed cases of Zika virus infection in Yucatan State and cases in Mexico. B) Cases of Zika virus infection among 115 pregnant women enrolled in study in 3 areas of Yucatan State.

If one considers the probability of acquiring Zika virus infection in relation to the moment of conception, those women who conceived during 2016 during epidemiologic weeks 13–40 had a statistically significant increased risk for acquiring the infection during pregnancy than for women who conceived during epidemiologic weeks 41–52 of 2016 and 1–12 of 2017 (odds ratio 5.86; p<0.001). For those patients who were positive at the time of enrollment, it was not possible to identify precisely when they became infected. In patients who were detected infected during follow-up, 0 became infected in the first trimester, 5 in the second trimester, and 5 in the third trimester. The average age of pregnant women in the study was 25 years, and we found no major differences in age distribution between Zika virus–positive and Zika virus–negative mothers (Table 3). We also found no difference in Zika virus infection for women of different socioeconomic status or between women residing in urban or rural areas (Table 3).

**Table 3:** PCR results for Zika virus infection of clinical samples from pregnant women who showed development of infection during follow-up*

Variable	No. (%)			p value†	Odds ratio
	Zika virus–positive, n = 36	Zika virus–negative, n = 79	Total, n = 115		
Age group, y					
15–19	7 (19)	19 (24)	26 (23)	0.639	0.76
20–29	20 (56)	42 (53)	62 (54)	0.843	1.1
30–49	9 (25)	18 (23)	27 (23)	0.815	1.13
Socioeconomic level‡					
1	4 (11)	3 (4)	7 (6)	0.12	3
2	13 (36)	42 (53)	55 (48)	0.09	0.4
3	13 (36)	24 (30)	37 (32)	0.54	1.2
4	6 (17)	10 (13)	16 (14)	0.56	1.3
Urban residency	33 (92)	70 (90)	103 (90)	0.78	1.25
Contacts tested positive					
Family members	3 (12)	3 (5)	7§	0.3	2.7
Partner	0	2 (3)	2¶	1	1.4
Working outside home	18 (50)	19 (24)	37 (32)	0.007	3.15
GW at admission to cohort, trimester					
5–13, first	8 (22)	22 (28)	30 (26)	NA	NA
14–27, second	23 (64)	39 (49)	62 (53)	NA	NA
28–40, third	5 (14)	18 (23)	23 (20)	NA	NA
LMD/EW#					
1–12	5 (15)	22 (33)	27 (27)	0.093	0.373
13–28	15 (45)	15 (22)	30 (30)	0.021	2.9
29–40	11 (33)	11 (16)	22 (22)	0.07	2.6
41–52	2 (6)	19 (28)	21 (21)	0.017	0.166
13–40/12–41	NA	NA	NA	<0.0001	5.86
Symptomatic	23 (64)	10 (13)	33 (28)	<0.0001	12.2
Exanthema	23 (100)	10 (100)	33 (100)	<0.0001	12.2
Conjunctival hyperemia	17 (73)	3 (30)	20 (60)	<0.0001	22.6
Arthralgia	15 (65)	3 (30)	18 (55)	<0.0001	18.1
Pruritus	11 (48)	0	11 (33)	<0.0001	NA
Headache	9 (39)	2 (20)	11 (33)	<0.0001	12.8
Retro-orbital pain	9 (39)	0	9 (27)	<0.0001	NA
Joint edema	7 (30)	1 (10)	8 (24)	0.001	18.8
Myalgia	6 (26)	1 (10)	7 (21)	0.004	15.6
Fever	4 (17)	1 (10)	5 (15)	0.033	9.7
Diarrhea	3 (13)	0	3 (9)	0.029	NA
Odynophagia	2 (9)	0	2 (6)	0.096	NA
Cough	2 (9)	0	2 (6)	0.096	NA
Congested	2 (9)	0	2 (6)	0.096	NA
Nausea	2 (9)	1 (10)	3 (9)	0.230	4.5
Vomiting	1 (4)	0	1 (3)	0.313	NA
Petechia	1 (4)	0	1 (3)	0.313	NA
Gingival bleeding	1 (4)	0	1 (3)	0.313	NA

*Initial samples for all patients showed negative results. EW, epidemiologic week; GW, gestational week; LMD, last menstruation date; NA, not applicable; T, trimester. †By Fisher exact test. ‡1, income insufficient to cover basic needs; 2, income just covers basic needs; 3, income for basic needs is met and includes certain recreational activities; 4, income is sufficient for recreational activities and luxuries. §90 studied cases. ¶87 studied cases. #>100 patients with LMD data.

More than half (64%) of the women had >1 sign or symptom compatible with acute infection (Table 3). We found that headache, retro-orbital pain, arthralgia, conjunctival hyperemia, joint edema, exanthema, and pruritus, each had a strong association with Zika virus infection (Table 3). If we considered separately only those objective signs that showed a strong association (conjunctival hyperemia, joint edema, and exanthema), we found that 7 Zika virus–positive had all 3 signs and that none of the Zika virus–negative patients had these 3 signs. All Zika virus–positive patients who had joint edema also had exanthema and conjunctival hyperemia (Table 3). One Zika virus–negative patient had joint edema associated with exanthema but without conjunctival hyperemia.

A total of 17 Zika virus–positive patients had conjunctival injection, which was present in only 3 Zika virus–negative patients. For these 17 patients, this infection was associated with exanthema, and for 7 patients, this infection was associated with joint edema. Six patients had only exanthema. For subjective but unusual symptoms, such as retro-orbital pain (9 patients) and pruritus (11 patients), we observed that all but 1 patient with retro-orbital pain also had conjunctival hyperemia, and all had exanthema. Of patients with pruritus, all had exanthema, 10 had conjunctival hyperemia, and 6 had joint edema. The most frequent clinical findings among Zika virus–positive women were exanthema, arthralgia, and conjunctival hyperemia. Headache, retro-orbital pain, joint edema, and pruritus were the most specific signs and symptoms, but these symptoms had low sensitivity (Table 4). The proportion of symptomatic Zika virus–positive patients did not vary between cities. We did not observe hemorrhagic or systemic complications in any patient.

**Table 4:** Statistical values of clinical variables for pregnant women infected with Zika virus, Yucatan, Mexico

Clinical variable	Positive predictive value, %	Negative predictive value, %	Diagnostic accuracy, %
Exanthema	70	84	80
Conjunctival hyperemia	85	80	80
Arthralgia	83	78	79
Itching	100	76	78
Headache	82	74	75
Retro-orbital pain	100	75	77
Joint edema	88	73	74

At the time of this study, all pregnancies were complete. Of these pregnancies, 3% were preterm, 2 for Zika virus–negative mothers and 1 for a Zika virus–positive mother (Table 5). Two fetal losses (2 in the first trimester and 1 in the third trimester) occurred among Zika virus–negative mothers. No newborns or products of conception were positive for Zika virus by virologic tests. We determined APGAR scores and percentiles of head circumference (Table 4). A Wilcoxon signed-rank test showed no significant difference between head circumference of babies from Zika virus–positive mothers and Zika virus–negative mothers (W = 213; p = 0.82). One newborn from a Zika virus–positive mother died during the first days of life because of gastroschisis. Other complications occurred among newborns but were nonspecific with regards to Zika virus infection status of the mothers (Table 4). Regarding the follow-up of the infants, although it is still in progress, no anomalies potentially related to Zika virus infection have been detected. Zika virus–positive mothers of 5 babies and Zika virus–negative mothers of 10 babies voluntarily withdrew from the study after delivery; this loss represented 13% of the cohort and was distributed proportionally between the 2 groups.

**Table 5:** Outcomes of pregnancy and for newborn children born to Zika virus–infected and –noninfected mothers, Yucatan, Mexico*

Outcome	Zika virus–positive mothers, n = 31	Zika virus–negative mothers, n = 69	Total, n = 100	p value†	Odds ratio
Pregnancy‡					
Live births	NA	NA	NA	NA	NA
Term	30 (97)	67 (97)	NA	0.90	0.89
Preterm	1 (3)	2 (3)	NA	0.90	1.10
Fetal loss, trimester					
First	0	2 (2)	2 (2)	NA	NA
Second	0	0	0	NA	NA
Third	0	1 (1)	1 (1)	NA	NA
Newborn					
APGAR score, 1 min, median (range)	7.9 (4–9)	8 (6–9)	NA	NA	NA
APGAR score, 5 min, median (range)	8.8 (5–9)	8.9 (8–9)	NA	NA	NA
Head circumference, median (range), cm	33.99 (29–36)	33.46 (29–35)	NA	0.82§	NA
PCR Zika virus–positive	0	0	0	NA	NA
Death or neonatal complications¶	5 (16)	3 (5)	8 (10)	0.06	4.20
Hyperbilirubinemia	1 (3)	2 (4)	3 (4)	0.90	1.10
Intrauterine growth retardation	0	2 (4)	2 (2)	0.47	NA
Syphilis	0	1 (2)	1 (1)	0.69	NA
Gastroschisis	1 (3)	0	1 (1)	0.31	NA
Erythema toxicity	2 (6)	0	2 (2)	0.09	NA
Microcephaly	0	1 (2)	1 (1)	0.69	NA
Anemia	0	1 (2)	1 (1)	0.69	NA

*Values are no. (%) unless otherwise indicated. NA, not applicable. †By Fisher exact test. ‡Loss of follow-up for 5 Zika virus–positive mothers and for 10 Zika virus–negative mothers. §By Wilcoxon signed-rank test. ¶Only 1 newborn death was observed and was from a Zika virus–positive mother.

## Discussion

Yucatan State in Mexico, where 84% of the population resides in urban areas, has been a hotspot for *Aedes* mosquitoborne diseases for many decades. After introduction of Zika virus, routine measures to avoid vector propagation (e.g., ultra-low volume spraying indoors and outdoors in areas where symptomatic cases were reported, community education), as well as personal protection against mosquito bites, were implemented by the regional government. Such interventions failed to contain Zika virus propagation (*24*) and were not directed toward pregnant women. The detailed evaluation of a cohort of pregnant women who were positive for Zika virus shortly before conception or who became infected during their pregnancy provided no evidence of vertical transmission to the fetus or fetal malformations directly attributed to Zika virus. Nonetheless, our evaluation of this cohort documented useful symptomology and demographic trends of Zika virus infection in pregnant women in a poorly studied area to which dengue virus and other flaviviruses are endemic.

We showed by univariate analyses that the most sensitive clinical sign was exanthema, but it was also the least specific. Conjunctival hyperemia, joint edema, and exanthema was the combination with the highest level of specificity. Given the cocirculation of Zika virus with other arboviruses in the region with which it shares common clinical characteristics (exanthema, headache, arthralgia), it is expected that the specificity, positive predictive value, and diagnostic accuracy will decrease in relation to the differential diagnosis. The absence of fever, as well as the presence of exanthema, with or without other signs and symptoms, should alert the primary health system to suspect Zika virus infection in pregnant women at any time during the evolution of their pregnancy.

The proportion of symptomatic cases observed in our cohort can be an expression of the bias in our enrollment strategy because patients referred by physicians from primary care facilities are more likely to be positive for Zika virus infection and symptomatic. During the study period, active circulation of chikungunya virus and dengue virus was reported in the area. Because our samples were not tested for these virus infections, the chance of a co-infection cannot be ruled out. This finding represents a major limitation when we analyzed the clinical approach to orient diagnosis.

The risk for congenital disease among Zika virus–infected pregnant women has been estimated to be 1%–13% (*25**–**28*), and this rate increases when the maternal infection occurs during the first and second trimesters (*29**,**30*). Although we did not detect direct evidence of congenital transmission by testing with RT-PCR, development of abnormalities attributable to Zika virus infection could still occur during infancy (*31*). The low number of pregnant women enrolled in this study could have precluded detection of congenital infection. Alternately, the absence of overt congenital Zika virus infection in this small cohort could reflect the relative rarity of this condition, as observed in other countries (*7**,**9**,**32**,**33*). In addition, the limitations to implement serologic tests because cross-reactivity with other flavivirus (*34**–**36*) could have masked laboratory confirmation of Zika virus infection for patients after the waning of virus presence in fluids or tissues. The lack of clinical manifestations at birth does not eliminate the possibility of congenital disease, as reported (*37*,*38*).

There is recognized discordance in Zika virus detection on concurrent blood and urine samples (*39**–**41*). Urine samples are transiently positive after virus is detected in blood. Urine samples alone were insufficient in detecting 16 cases. A total of 43% of Zika virus–positive patients had a viremia for >4 weeks and 15% for >8 weeks. This prolonged viremia, which is unusual for other arboviruses, has been reported in pregnant women in other studies (*42**,**43*). The role of this prolonged viremia on pathogenesis of congenital diseases or dissemination of the infection is unclear.

Women who work outside had an increased risk for contracting the infection, potentially reflecting differential exposure to *Aedes aegypti* mosquitoes at locations other than their home (*44**,**45*); the highest incidence of pregnancies in women 20–29 years of age is consistent with results of another case series (*32*). We have not observed major differences in Zika virus infection in different age groups. We have observed that the highest proportion of Zika virus–positive women with symptomatic disease was among women 20–29 years of age, which is different from other studies that reported the highest ratio of symptomatic disease among women >30 years of age (*46**,**47*).

Although knowledge of clinical manifestations, natural history, and epidemiology of Zika virus in the Americas is incipient, the clinical–epidemiologic scenario involving severe congenital disease that was manifested initially in Brazil has not been observed at the same magnitude in other countries. Our prospective study of a cohort of pregnant women in Yucatan, Mexico, showed the value of active surveillance in early detection of infections and point to the limited predictive value of symptoms in areas where Zika virus cocirculates with other flaviviruses. In our study of 115 pregnant women with active or recent Zika virus infections, we found no evidence of congenital Zika virus disease.
